# A Fluorescence Imaging-Based 3D Analysis Pipeline for Mouse Trigeminal Ganglion Neurons

**DOI:** 10.3390/bios16060333

**Published:** 2026-06-11

**Authors:** Jiajia Wang, Xinyu Yuan, Jianchao Zhang, Jingyi Che, Xiaojun Wang

**Affiliations:** State Key Laboratory of Digital Medical Engineering, Key Laboratory of Biomedical Engineering of Hainan Province, School of Biomedical Engineering, Hainan University, Sanya 572025, China

**Keywords:** fluorescence imaging, 3D reconstruction, bioimaging, fMOST, propidium iodide staining, retrograde viral labeling, trigeminal ganglion

## Abstract

As the primary peripheral relay station for vibrissal tactile information, the trigeminal ganglion (TG) features heterogeneous three-dimensional (3D) cytoarchitecture that eludes full characterization using conventional two-dimensional methodologies. A high-resolution 3D imaging and reconstruction pipeline is thus required to unveil TG structural organization and define the spatial framework of target-related sensory neurons. Herein, we established a fluorescence micro-optical sectioning tomography (fMOST)-based workflow for 3D cytoarchitectural mapping of TG anatomy and validated its utility for profiling the distributions of TG neurons innervating vibrissae via single-axon tracing. fMOST imaging coupled with propidium iodide (PI) staining was applied to acquire whole-head anatomical data encompassing the vibrissae and the TG at cellular resolution. Based on clearly resolved cellular morphology and the spatial distribution of neuronal somata, we delineated the soma distribution of TG neurons and revealed a spatially heterogeneous 3D organization pattern, from which we operationally defined two anatomically distinct subdomains: the neuronal soma-rich region (NSRR) and the fiber-rich region (FRR). Furthermore, with retrograde viral/genetic labeling combined with neuronal tracing, TG neurons innervating the C2, D3, and δ vibrissae were observed in both NSRR and FRR, showing partially overlapping yet spatially biased distributions consistent with previous population-level observations of vibrissa-row-dependent topography. Notably, TG neurons innervating the δ vibrissa occupied a comparatively broader spatial extent along the anteroposterior plane in our dataset. Overall, this study facilitates an in-depth mechanistic and anatomical understanding of TG cytoarchitectural organization and underlying functional mechanisms.

## 1. Introduction

The rodent vibrissal system is a highly specialized tactile sensory system in which mechanical signals detected by the vibrissae are conveyed by primary sensory neurons in the trigeminal ganglion (TG), relayed through the brainstem and thalamus, and ultimately processed in the primary somatosensory cortex (S1) [1]. Within this sensory system, topographic organization corresponding to the vibrissal arrangement pattern is formed at each level of the central nervous system (CNS). Each vibrissa is associated with a specific neuronal cluster at each level of the CNS [2,3]. In contrast, the TG is a peripheral ganglion without an archetypal or laminar organization, showing structural heterogeneity, and its internal cytoarchitecture is characterized by heterogeneous distributions of neuronal somata, nerve fibers, satellite glial cells (SGCs), and Schwann cells (SCs) [4,5,6]. Because of this structural heterogeneity, high-resolution three-dimensional (3D) imaging is needed to characterize TG cytoarchitecture and to provide a spatial framework for localizing TG neurons innervating vibrissae.

Previous studies of TG cytoarchitecture have relied largely on two-dimensional (2D) histological sections [7,8]. This conventional approach limits comprehensive understanding of the 3D cytoarchitecture and continuous tissue organization of the TG. Studies of TG neurons innervating vibrissae have relied largely on retrograde tracing techniques [9,10,11] and electrophysiological approaches [12,13,14]. However, many of these studies analyzed labeled neuronal populations without explicitly incorporating the internal cytoarchitectural heterogeneity of the TG. Three-dimensional (3D) imaging is important because it addresses several inherent limitations of conventional 2D microscopic analysis, such as time-consuming sectioning procedures, tissue damage resulting from physical sectioning, and limited spatial continuity [15,16].

Tissue-clearing-based 3D imaging has provided an important approach for visualizing TG neurons [17,18]. Although such approaches have enabled volumetric visualization of TG neurons, they may be affected by tissue deformation, uneven clearing or labeling, and challenges in preserving fine spatial relationships across large samples. Fluorescence micro-optical sectioning tomography (fMOST) [19], which combines serial imaging with physical sectioning, provides an alternative strategy for continuous high-resolution imaging of large-volume tissues without relying on tissue clearing. In addition, the resin-embedded state of the sample ensures structural stability, preventing deformation during sectioning and providing an inherent advantage for image registration, making this approach suitable for high-precision 3D reconstruction of the tissue. Therefore, fMOST is suitable for acquiring 3D datasets that preserve the spatial continuity among the mystacial pad, TG, and brain.

In this study, we established a fluorescence micro-optical sectioning tomography (fMOST)-based workflow for 3D cytoarchitectural mapping of TG anatomy and validated its utility for profiling the distributions of TG neurons innervating vibrissae via single-axon tracing. fMOST imaging combined with propidium iodide (PI) staining was applied to acquire whole-head anatomical data encompassing the vibrissae and the TG at cellular resolution and to visualize the cytoarchitectural organization of the mouse TG. Based on clearly resolved cellular morphology and the spatial distribution and packing density of neuronal somata in this PI-stained structural dataset, we reconstructed the neuronal soma distribution region and operationally defined two anatomically distinct subdomains: the neuronal soma-rich region (NSRR) and the fiber-rich region (FRR). These regions were used as a practical anatomical framework for subsequent spatial analysis, rather than as statistically validated subdivisions across animals. We further performed local neuronal soma enrichment analysis to provide a quantitative measure of TG cytoarchitectural heterogeneity. Previous studies have shown that TG neurons innervating vibrissae differ more prominently by row than by arc, with neurons within the same row exhibiting less spatial separation than those across different rows [13,20,21]. Therefore, we selected two representative regular vibrissae from non-neighboring ventral rows, the C2 and D3 vibrissae. The δ vibrissa was also included because it is a large caudally located straddler vibrissa with a distinct anatomical position relative to regular grid vibrissae. With retrograde viral/genetic labeling combined with neuronal tracing, we reconstructed TG neurons innervating the C2, D3, and δ vibrissae by tracing labeled peripheral axons and somata. We also reconstructed endogenous autofluorescence within the TG, which likely included lipofuscin-like signals, as a macroscopic background map of the neuronal population. This background map provided an internal spatial reference for localizing specifically labeled TG neurons innervating vibrissae.

Using this fMOST-based workflow, we aimed to establish a practical 3D method for TG cytoarchitectural mapping and to validate its utility for profiling the distributions of TG neurons innervating vibrissae. In the analyzed dataset, the distributions of reconstructed neurons were partially overlapping and broadly consistent with previous population-level observations of vibrissa-row-dependent topography, whereas neurons innervating the δ vibrissa occupied a relatively broader spatial range along the anteroposterior plane.

## 2. Materials and Methods

### 2.1. Animals

Adult C57BL/6J mice and Ai14 reporter mice (JAX, stock number: 007908) aged 2 to 8 months were used in this study. C57BL/6J mice were purchased from SPF (Beijing) Biotechnology Co., Ltd. (Beijing, China) Ai14 mice were purchased from Jackson Laboratory (Bar Harbor, ME, USA). For the 3D segmentation of the TG subregions, the C57BL/6J mouse was used for fMOST imaging with PI staining. For the imaging and reconstruction of the TG neurons innervating vibrissae, the Ai14 mouse was used for sparse labeling with virus injection, and all neuronal reconstructions in this study were derived from the same animal. Mice were housed under a 12 h light/dark cycle with ad libitum access to food and water. All animal experiments were performed in accordance with the procedures approved by the Hainan University Animal Welfare and Ethics Committee.

### 2.2. Virus Injection

Mice were deeply anesthetized using isoflurane (RWD Life Science Co., Ltd., Shenzhen, China), and after anesthesia, ophthalmic ointment was applied to both eyes. Then the mouse was positioned in a lateral position with the mouth facing outward and the face exposed. A tubular tool with a card slot structure was fabricated using 3D printing technology to facilitate subsequent fixation of hemostats and completion of injection procedures. Hemostats were used to clamp the mouse vibrissae, with the tips of the vibrissae protruding outward to form a small bump on the skin surface of the mystacial pad. A glass injection needle was inserted into the dorsal side of this small bump for viral delivery. We injected 600 nL of the AAV2/2Retro-hSyn-Cre-WPRE-PA (1.39 × 10^13^ genome copies mL^−1^; Shanghai Taitool Bioscience Co., Ltd., Shanghai, China) into the follicle–sinus complexes (FSCs) of the mystacial pad at a flow rate of 1 nL/s. Specifically, 200 nL of the virus was administered at FSC depths of 0.6, 0.4, and 0.2 mm from the surface of the mystacial pad. Following virus injection, mice were maintained on a heating pad to keep their core body temperature at 37 °C. Mice were then returned to their home cages and allowed 21 days for viral transgene expression prior to tissue collection and imaging.

### 2.3. Histology and Microscopy

The C57BL/6J mouse was deeply anesthetized with isoflurane (RWD Life Science Co., Ltd., Shenzhen, China) and subsequently intracardially perfused with 0.01 M phosphate-buffered saline (PBS; Beijing Labgic Technology Co., Ltd., Beijing, China), followed by 4% paraformaldehyde (PFA; Sangon Biotech (Shanghai) Co., Ltd., Shanghai, China). The trigeminal ganglion was excised and postfixed in 4% PFA in PBS at 4 °C overnight and then washed in PBS before sectioning. For immunohistochemistry, the trigeminal ganglion was sectioned into 50 µm thick slices using a vibrating microtome (VT1000S, Leica Biosystems Nussloch GmbH, Nussloch, Germany). Before staining, sections were washed 3 × 10 min in PBS. Sections of interest were processed as follows: blocking in 0.01 M PBS containing 10% (*w*/*v*) normal goat serum (NGS) and 0.3% (*v*/*v*) Triton X-100 (Sigma-Aldrich, St. Louis, MO, USA) for 1 h at room temperature; incubation with primary antibody (mouse anti-NeuN, 1:800, Sigma-Aldrich, St. Louis, MO, USA; cat. no. MAB377) at 4 °C for 12 h; washes with 3 × 10 min in PBS, followed by incubation with fluorochrome-conjugated secondary antibody (Alexa Fluor 488-conjugated goat anti-mouse IgG (H+L), 1:800, Abcam plc, Cambridge, UK; cat. no. ab150113) at 4 °C for 3 h; and final washes with 3 × 10 min in PBS. All antibodies were diluted in the aforementioned blocking solution.

The sections were soaked in PBS with 1 µg/mL DAPI (4’,6-diamidino-2-phenylindole; Thermo Fisher Scientific, Waltham, MA, USA) for 1 min and then washed in PBS for 2 min. Finally, the sections were mounted in 50% glycerol (vol/vol) and imaged using a confocal microscope (Nikon Corporation, Tokyo, Japan) equipped with 20× (NA 0.75) and 60× (NA 1.42) objectives.

### 2.4. fMOST Imaging

(1) Sample Preparation

After transcardiac perfusion with 0.01 M PBS and 4% PFA, mouse heads were excised and postfixed in 4% PFA for 24 h at 4 °C. After fixation, the mouse heads were washed with 0.01 M PBS at 4 °C to remove residual PFA.

(2) Resin embedding

The resin-embedding protocol was used to maintain structural continuity and to preserve tdTomato fluorescence generated by retrograde viral tracing in Ai14 reporter mice.

Large-volume mouse heads were embedded with optimized glycidyl methacrylate (GMA; Sigma-Aldrich, St. Louis, MO, USA) resin. Samples were dehydrated in a graded ethanol series (50% ethanol for 12 h; 75% ethanol for 4 h twice; 95% ethanol for 4 h; 95% ethanol for 12 h; all steps performed at 4 °C) (Xilong Scientific Co., Ltd., Shantou, China), followed by infiltration with a graded GMA resin series (50% resin for 3 h; 75% resin for 3 h twice; 100% GMA resin for 24 h twice; each step at 4 °C), then polymerized at 38 °C for 1 h, 38 °C for 4 h, 40 °C for 30 min, 40 °C for 8 h. The 100% GMA resin composition was 75% GMA, 15% butyl methacrylate (BMA; Shanghai Macklin Biochemical Co., Ltd., Shanghai, China), 10% triethylene glycol dimethacrylate (TEDGMA; Shanghai Macklin Biochemical Co., Ltd., Shanghai, China), and 0.1% 2,2’-azobis(2-methylpropionitrile) (ABVN; Shanghai Titan Scientific Co., Ltd., Shanghai, China). The 50%, 75%, and 95% GMA resin solutions (*w*/*w*) were prepared by diluting 100% GMA resin with 95% ethanol.

(3) Imaging

Precise imaging of mouse heads for cytoarchitectures of TG was performed using an optimized three-color TDI-fMOST system [22,23] (BioMapping 5000, Wuhan OE-Bio Co., Ltd., Wuhan, China) equipped with a 60× water-immersion objective (1.0 NA, Nikon Corporation, Tokyo, Japan) and three TDI CCDs (C10000-C01, Hamamatsu Photonics K.K., Hamamatsu, Japan). Samples for structural imaging (C57BL/6J mice) were imaged at a voxel resolution of 0.233 × 0.233 × 1 μm and immersed in a 2 μg/mL propidium iodide (PI; Thermo Fisher Scientific, Waltham, MA, USA) solution during imaging (excitation laser: 561 nm).

Virus/genetically labeled samples (Ai14 mice; tdTomato was expressed) were soaked in a 0.05 M Na_2_CO_3_ (Xilong Scientific Co., Ltd., Shantou, China) solution and imaged at a voxel resolution of 0.325 × 0.325 × 1 μm using line illumination modulation (LiMo; HD-fMOST system) during imaging (excitation laser: 561 nm) [24]. The fMOST imaging workflow was as follows: the stage was moved so that the objective scanned the sample surface for initial imaging, followed by removal of the imaged layer using a diamond knife, and repeated cycles of surface imaging and sectioning were performed until the entire tissue volume was imaged, yielding complete and continuous datasets.

### 2.5. Image Preprocessing

All image preprocessing was implemented following standard protocols for established fMOST pipelines [22,23,24]. Raw images were preprocessed using a custom C++ program (C++11) for brightness nonuniformity correction and seamless image stitching to generate coronal image sequences. Subsequently, image optimization was performed to improve background uniformity, reduce periodic noise, and enhance contrast. These procedures included background correction, noise reduction, and contrast enhancement.

### 2.6. 3D Reconstruction

(1) 3D segmentation of the TG subregions

To reconstruct the 3D distribution of TG neuronal somata, based on the coordinate data of the TG in the cytoarchitecture channel, separate image sets around the TG were extracted using preprocessor software. Using Fiji (ImageJ 1.54f), the extracted images were combined into a new image set by saving every four images as a group. This new image set was opened in Amira software (version 6.1.1, FEI). In the “Segmentation” module, the “Label Field” function was selected, and the brush tool was used to delineate the contours of the structures (the TG contour and the distribution regions of somata) in each 2D image. After all images were annotated, the planar data were integrated. Finally, the labeled planes were rendered as 3D structures, allowing the planar labeled data to be reconstructed into three-dimensional structures. Different structures were represented using distinct labels to ensure they could be separated after three-dimensional reconstruction. To reconstruct the 3D distribution of autofluorescence, based on the coordinate data of the TG in the red channel, a separate image set around the trigeminal ganglion was extracted using preprocessor software, and the images were subjected to maximum-intensity projection over consecutive non-overlapping 10 μm stacks to generate a new image set. The subsequent segmentation and reconstruction steps were the same as those described above.

(2) Single-neuron reconstruction

To perform 3D reconstruction of TG neurons innervating vibrissae, preprocessed large-volume datasets were converted into TDat format [25]. Data were then imported into Amira software (version 6.1.1), where image stacks were viewed interactively, and neurons were traced using the Line Editor tool. All tracing data for neuron reconstruction were saved in Standardized Wiring Diagram (SWC) format. Due to the limitations in imaging and labeling, it was not possible to completely reconstruct the axon terminals of the labeled TG neurons within the FSC. Therefore, we chose to start from the deep vibrissal nerve (DVN) that innervates a specific FSC and reconstruct the portion of the DVN just before it enters the FSC, thereby ensuring that the reconstructed neurons were those associated with the target vibrissa. For soma reconstruction, images were adjusted to a specific contrast to show the cell surface. The reconstruction was conducted with the Segmentation Editor. To reduce reconstruction bias, no further contrast adjustment was performed during the delineation of the cell body boundaries.

### 2.7. Local Neuronal Soma Enrichment Analysis

Local neuronal soma enrichment analysis was performed using the reconstructed TG mask and neuronal soma distribution binary mask. The TG volume was divided into 3D spatial bins of 20 × 20 × 20 μm. For each bin, the local soma fraction was calculated as the number of soma mask voxels divided by the number of TG mask voxels within that bin. The local neuronal soma enrichment factor was then calculated by normalizing the local soma fraction to the average soma fraction across the entire TG:

$$EF_{i}=\frac{N_{\mathrm{soma},i}/N_{\mathrm{TG},i}}{N_{\mathrm{soma},\mathrm{total}}/N_{\mathrm{TG},\mathrm{total}}}$$

where ($EF_{i}$) represents the neuronal soma enrichment factor of the (i)-th spatial bin; ($N_{\mathrm{soma},i}$) and ($N_{\mathrm{TG},i}$) represent the numbers of soma mask voxels and TG mask voxels within that bin, respectively; and ($N_{\mathrm{soma},\mathrm{total}}$) and ($N_{\mathrm{TG},\mathrm{total}}$) represent the corresponding voxel numbers across the entire TG. An enrichment factor greater than 1 indicates a higher local soma fraction than the TG-wide average, whereas a value less than 1 indicates a lower local soma fraction.

### 2.8. Statistical Analysis

Morphological and spatial analyses were performed in this study. No inferential statistical tests were conducted because all reconstructed TG neurons innervating vibrissae were derived from a single mouse.

## 3. Results

### 3.1. fMOST-Based Workflow for 3D TG Cytoarchitectural Analysis and Single-Neuron Reconstruction of TG Neurons Innervating Vibrissae

**Figure 1:**
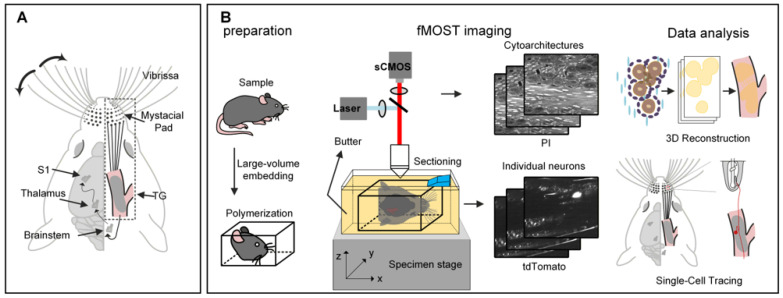
Overview of the fMOST-based 3D imaging and reconstruction workflow for analysis of the mouse TG: (**A**) Schematic of tactile signal transmission in the mouse. (**B**) Procedure for sample preparation, fMOST imaging, and data analysis.

As the first station for transmitting tactile information from the periphery to the CNS, the TG is the focus of this study (Figure 1A). We established a fluorescence micro-optical sectioning tomography (fMOST)-based workflow for 3D cytoarchitectural mapping of TG anatomy and profiling the distributions of TG neurons innervating vibrissae.

To perform 3D cytoarchitectural mapping of TG anatomy and profile TG neurons innervating selected vibrissae, we combined large-volume embedding, fMOST imaging, and data processing (Figure 1). The experimental procedure was as follows: (1) The mouse head was embedded for large-volume imaging, and fMOST combined with PI staining was used to acquire cellular-resolution 3D imaging data of the embedded tissue. Based on these data, we identified neuronal somata and reconstructed the neuronal soma distribution region. (2) A subset of TG neurons innervating selected vibrissae was labeled by injecting AAV2/2Retro-hSyn-Cre-WPRE-PA into FSCs of the mystacial pad of Ai14 mice. After sufficient viral expression, large-volume embedding was performed on the mouse head tissue, and 3D imaging data of the embedded tissue were acquired using fMOST. We then reconstructed TG neurons innervating the C2, D3, and δ vibrissae within the TG by tracing labeled peripheral axons and somata. Endogenous autofluorescence within the TG was also reconstructed as a macroscopic neuronal background map to provide an internal spatial reference for localizing labeled neurons.

### 3.2. Identification of TG Neuronal Somata Using fMOST Combined with PI Staining

To preserve the structural continuity among the TG, the mystacial pad, and the brain, and to perform 3D cytoarchitectural information of the TG, we collected a 3D dataset of the mouse head using the fMOST-based workflow described above (Figure 1). This PI-stained structural dataset contained the brain, the TG, and the mystacial pad from a C57BL/6J mouse within a continuous 3D imaging volume.

**Figure 2:**
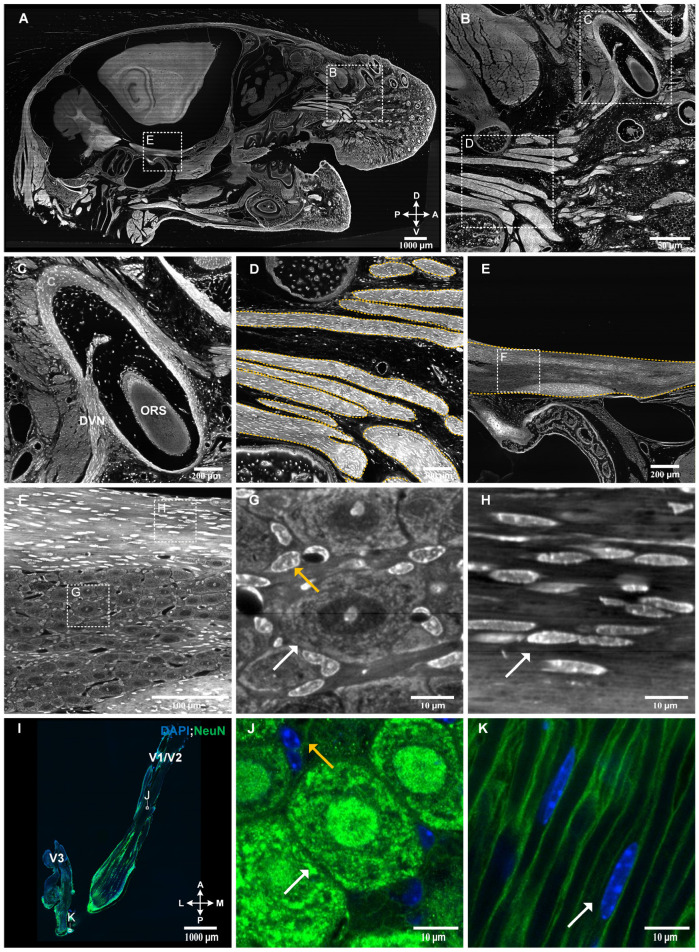
fMOST imaging combined with PI staining of the mouse head and morphological identification of TG neuronal somata: (**A**,**B**) Sectional images of the mouse head acquired using fMOST imaging combined with PI staining (red; voxel resolution: 0.233 µm × 0.233 µm × 1 µm), showing the structural continuity and enabling clear identification of the anatomical boundaries of head tissues. (**C**) Magnified view of the mystacial pad showing vibrissa-related structures, including the outer root sheath (ORS), collagenous capsule (C), and deep vibrissal nerve (DVN). (**D**) Magnified view showing the trigeminal nerves innervating the right mystacial pad; yellow circles outline the trigeminal nerves. (**E**) Magnified view showing the TG at the skull base; yellow dashed lines indicate the TG region. (**F**) Higher magnification showing cell morphologies. (**G**) Insets showing the somata of TG neurons (white arrows) and SGCs (yellow arrows). (**H**) Insets showing SCs (white arrows). (**I**) Staining results from independent TG samples (10× magnification). (**J**) Magnified view showing neuronal somata (white arrows) and SGCs (yellow arrows) (60× magnification). (**K**) Magnified view showing elongated oval SCs (white arrows) (60× magnification). A: anterior; P: posterior; D: dorsal; V: ventral; L: lateral; M: medial; V1: ophthalmic nerve; V2: maxillary nerve; V3: mandibular nerve; DVN: deep vibrissal nerve; C: collagenous capsule; ORS: outer root sheath. Scale bars: (**A**,**I**) 1000 μm, (**B**) 50 μm, (**C**–**E**) 200 μm, (**F**) 50 μm, and (**G**,**H**,**J**,**K**) 200 μm.

In the PI-stained cytoarchitecture channel, we could clearly identify the brain tissue, the TG, and the vibrissae (Figure 2A). Within the region of the right mystacial pad (Figure 2B), we could identify various structures closely related to vibrissae, such as the outer root sheath (ORS), collagenous capsule (C), and DVN, which transmits vibrissa-derived tactile information (Figure 2C), as well as the trigeminal nerve (Figure 2D). At the skull base (Figure 2A), we could identify the TG based on its anatomical location and structural characteristics (Figure 2E). These results indicate that fMOST imaging combined with PI staining can be used to acquire a continuous mouse head dataset that preserves the spatial relationship among the mystacial pad, TG, and brain.

Within the TG, we observed cells with distinct sizes, shapes, and packing densities (Figure 2E,F). TG neuronal somata were recognized based on their large cell bodies and characteristic morphological features, allowing reconstruction of the neuronal soma distribution region within the TG. To support the morphological identification of neuronal somata, we co-stained TG sections with NeuN (a neuronal marker) and the fluorescent dye DAPI, with DAPI used to morphologically identify the nuclei of neurons and glial cells (Figure 2I). We observed neurons with oval nuclei (Figure 2J), SGCs surrounding neurons (Figure 2J), and elongated oval SCs (Figure 2K). We found that neurons in the PI-stained sagittal sections exhibited morphological similarity to neurons stained with NeuN (Figure 2G); the morphologies of SGCs and SCs were similar to those of glial cells visualized by DAPI staining in sections (Figure 2H). These results support the feasibility of using fMOST imaging combined with PI staining to support cellular-resolution TG cytoarchitectural mapping and to identify neuronal somata based on morphological features.

### 3.3. 3D Cytoarchitectural Mapping and Local Enrichment Analysis of TG Neuronal Soma Distribution

**Figure 3:**
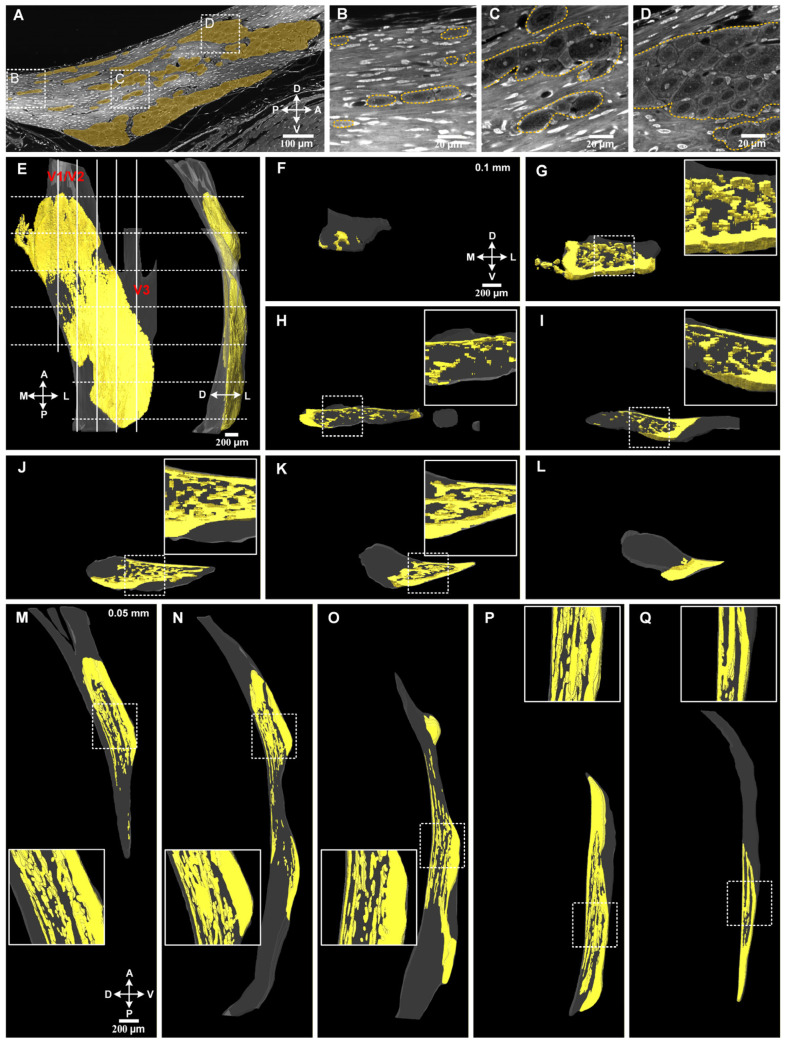
Distribution patterns of neuronal somata in the TG: (**A**) Sagittal section imaging of the TG, showing the TG contour and the distribution regions of somata; yellow circles outline the distribution regions of somata. (**B**–**D**) Local magnifications showing the distribution regions of somata and the shapes of somata. (**E**) The 3D reconstruction of the TG. White dashed lines and white solid lines in (**E**) correspond to the local 3D spatial distributions shown in (**F**–**L**) and (**M**–**Q**), respectively. Centered on each white dashed line and white solid line, the images show the 3D spatial distribution at the coronal plane level with a thickness of 0.1 mm and at the sagittal plane level with a thickness of 0.05 mm. (**F**–**L**) Local 3D spatial distribution results of the TG in the regions demarcated by white dashed lines in (**E**), showing 3D spatial distribution within each region. Insets are magnifications of the dashed boxed areas. (**M**–**Q**) Local 3D spatial distribution results of the TG in the regions demarcated by white solid lines in (**E**), showing 3D spatial distribution within each region. Insets are magnifications of the dashed boxed areas. Neuronal soma distribution regions are shown in yellow, and the tissue contour of the TG is shown in gray, providing an intuitive visualization of the 3D spatial distribution of somata within the TG. A: anterior; P: posterior; D: dorsal; V: ventral; L: lateral; M: medial; V1: ophthalmic nerve; V2: maxillary nerve; V3: mandibular nerve. Scale bars: (**A**) 100 μm, (**B**–**D**) 20 μm, and (**E**–**Q**) 200 μm.

To characterize the distribution and morphology of neuronal somata within the TG, we segmented the intact TG region from the complete 3D mouse head dataset and examined the spatial organization of neuronal somata. In 2D sagittal sections, we observed that neuronal somata formed discrete aggregates (Figure 3A), and somata within the aggregates were arranged in bands and clusters (Figure 3A), consistent with previous studies [7,8]. We observed that somata in the ventral region were more densely packed and visibly larger in size compared to those in the dorsal region, where somata were smaller and more distinctly separated (Figure 3A–D). Neuronal somata also showed diverse morphologies, including round, oval, polygonal, and elongated shapes (Figure 3B–D). We observed that elongated somata were predominantly localized to the dorsal region, whereas round, oval, and polygonal somata were primarily distributed in the ventral region.

To visualize the 3D distribution of neuronal somata within the TG, we segmented the distribution regions of neuronal somata from the PI-stained fMOST dataset using the Segmentation module of Amira software and performed 3D reconstruction. The reconstructed dataset revealed a spatially heterogeneous 3D organization pattern of neuronal somata. We observed that clusters of neuronal somata were densely aggregated in the anteromedial (Figure 3G,H), posterolateral (Figure 3K,L), and ventral (Figure 3G,I–L) regions of the TG, whereas somata were scattered in the dorsal region (Figure 3G–K). In addition, neuronal somata were arranged along the anteroposterior axis (Figure 3M–Q).

**Figure 4:**
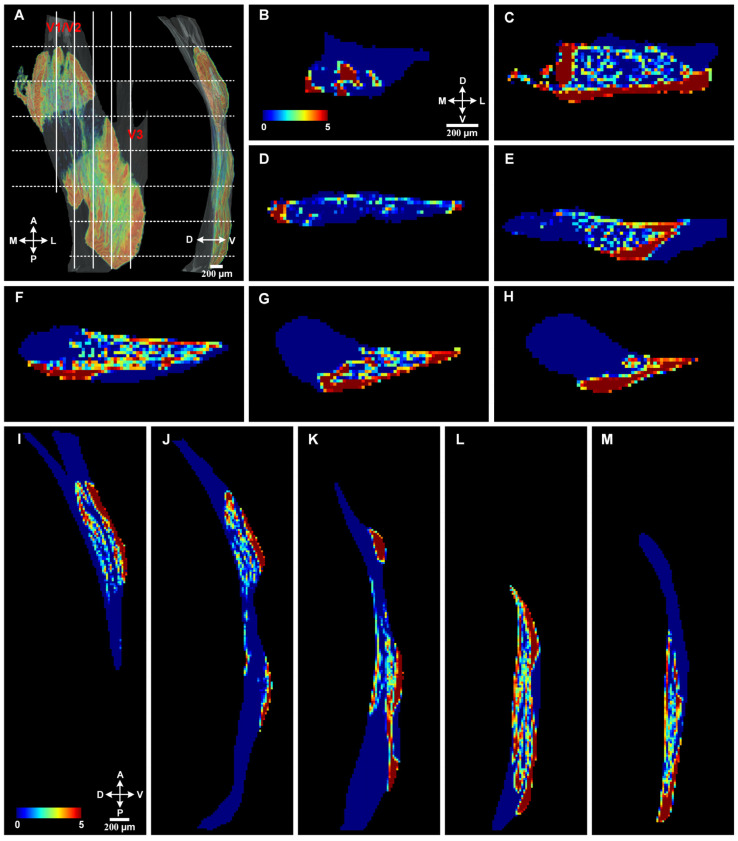
Local neuronal soma enrichment analysis of the TG based on the neuronal soma binary mask: (**A**) A 3D view of the local neuronal soma enrichment map within the TG. White dashed lines and white solid lines in (**A**) indicate the positions of the representative coronal and sagittal views shown in (**B**–**H**) and (**I**–**M**), respectively. (**B**–**H**) Representative coronal views of the local neuronal soma enrichment map at different anteroposterior levels. (**I**–**M**) Representative sagittal views of the local neuronal soma enrichment map at different mediolateral levels. The reconstructed TG volume was divided into 3D spatial bins, and each bin was colored according to its local neuronal soma enrichment factor. Warmer colors indicate higher local neuronal soma enrichment, whereas cooler colors indicate lower local enrichment. Black indicates the background outside the TG. The color scale represents the local neuronal soma enrichment factor. A: anterior; P: posterior; D: dorsal; V: ventral; L: lateral; M: medial; V1: ophthalmic nerve; V2: maxillary nerve; V3: mandibular nerve. Scale bars: 200 μm.

To further characterize the spatial heterogeneity of neuronal soma distribution, we divided the reconstructed TG volume into 3D spatial bins (20 × 20 × 20 μm) and calculated a local neuronal soma enrichment factor for each bin. Representative coronal and sagittal views of the local enrichment map showed that neuronal soma enrichment was not uniformly distributed across the TG, but varied across different local regions (Figure 4).

Based on the observed heterogeneity in soma density within this specimen, we operationally defined two anatomically distinct subdomains for subsequent analysis: a neuronal soma-rich region (NSRR) and the fiber-rich region (FRR). For the purpose of this study, we adopted a practical criterion: the peripheral, soma-dense regions of the TG were designated as NSRR, whereas the central, less soma-enriched region was designated as FRR. These subdomains were used as practical cytoarchitectural references rather than strictly segmented anatomical subdivisions.

### 3.4. Retrograde Labeling and Single-Neuron Reconstruction of TG Neurons Innervating Vibrissae

**Figure 5:**
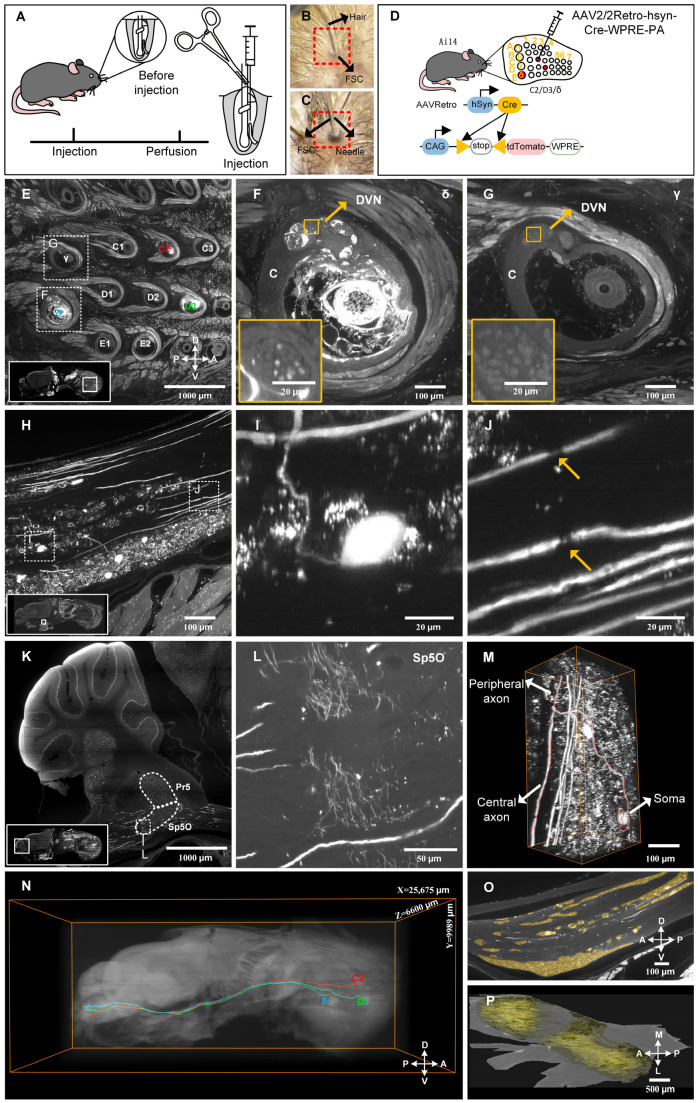
Retrograde labeling and single-neuron reconstruction of TG neurons innervating vibrissae: (**A**) Schematic diagram of the injection method for the mouse vibrissae. (**B**,**C**) Bright-field images of the mouse vibrissae showing vibrissal morphology before injection (**B**) and during injection (**C**). (**D**) Schematic diagram showing the principle of specific labeling of neurons innervating the vibrissae. (**E**) Section imaging of the mouse mystacial pad using fMOST; white boxes indicate the “injection site” (with fluorescent signals) and “non-injection site” (without fluorescent signals). (**F**) Local magnification showing the injection site; inset (lower left) is a magnified view of the yellow-boxed area; scale bar in insets: 20 µm. (**G**) Local magnification showing the non-injection site; inset (lower left) is a magnified view of the yellow-boxed area; scale bar in insets: 20 µm. (**H**) Section imaging of the ipsilateral TG using fMOST. (**I**) Local magnification showing a labeled neuronal soma (oval-shaped). (**J**) Local magnification showing labeled nerve fibers and nodes of Ranvier (yellow arrows). (**K**) Section imaging of the CNS using fMOST. (**L**) Local magnification showing that fluorescent signals were detected in the Sp5O. (**M**) Partial structural reconstruction of a labeled neuron, showing the soma and branches, as well as portions of central axons and peripheral axons. (**N**) Schematic diagram showing TG neurons innervating the C2, D3, and δ vibrissae by tracing from the peripheral axons. (**O**) Section imaging of the TG showing distribution of autofluorescence; yellow circles outline autofluorescent signals. (**P**) The 3D reconstruction of autofluorescence in the TG. A: anterior; P: posterior; D: dorsal; V: ventral; Pr5: the main or principal sensory nucleus; Sp5O: spinal trigeminal nucleus, oral part. Scale bars: (**E**,**K**) 1000 μm, (**F**–**H**,**M**,**O**) 100 μm, (**I**,**J**) 20 μm, (**L**) 50 μm, and (**P**) 500 μm.

To achieve the targeted retrograde labeling of TG neurons innervating the mouse vibrissae, we first lifted the target vibrissae (Figure 5A–C) and then injected AAV2/2Retro-hSyn-Cre-WPRE-PA into the FSCs of the selected vibrissae in Ai14 transgenic mice (Figure 5D). At 21 days post-injection, we obtained a dataset of the mouse head containing the mystacial pad, the TG, and the brain using large-volume embedding and the fMOST system.

We observed obvious tdTomato fluorescent signals at the three injection sites (C2 vibrissa, D3 vibrissa, and δ vibrissa) (Figure 5E). We further examined both injected and non-injected vibrissae and found fluorescent signals in the DVN of injected vibrissae, whereas no signals were detected in non-injected vibrissae (Figure 5F,G), supporting successful and localized retrograde labeling of vibrissa-related peripheral nerves. Subsequently, we analyzed fMOST images of the ipsilateral TG. We observed labeled neuronal somata and nerve fibers within the TG (Figure 5H). At high magnification, we observed that labeled neuronal somata were oval-shaped (Figure 5I), and nodes of Ranvier in nerve fibers were clearly identifiable (Figure 5J). Additionally, we detected tdTomato signals in the oral subnucleus of the spinal trigeminal nucleus (Sp5O) (Figure 5K,L), indicating that labeled TG neurons also projected to trigeminal sensory nuclei. These results indicate that specific labeling of TG neurons innervating the selected vibrissae was effectively achieved via our workflow.

To evaluate whether this dataset was suitable for profiling TG neurons innervating vibrissae via neuronal tracing, we performed single-neuron reconstruction of labeled neurons within the TG. We were able to trace and visualize TG neurons innervating the C2, D3, and δ vibrissae, including their soma, central axons, and peripheral axons (Figure 5M,N). This result indicates that the retrograde labeling dataset supports single-neuron profiling of TG neurons innervating vibrissae for subsequent spatial analysis.

To provide a macroscopic neuronal background map for the specifically labeled neurons, we segmented and reconstructed endogenous autofluorescence within the TG (Figure 5O,P). This autofluorescence map, while not a precise delineation of individual somata, provided a macroscopic neuronal background and served as an internal anatomical reference for localizing reconstructed TG neurons innervating vibrissae in subsequent analyses.

### 3.5. 3D Spatial Localization of TG Neurons Innervating the C2, D3, and δ Vibrissae

**Figure 6:**
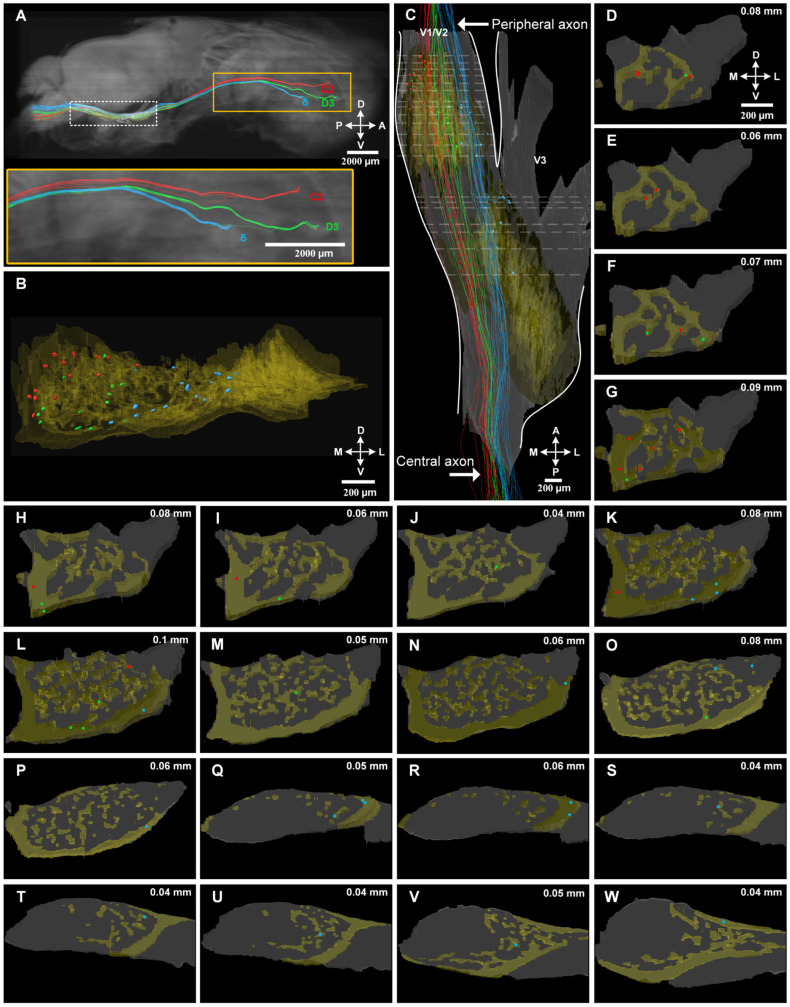
3D spatial localization of reconstructed TG neurons innervating the C2, D3, and δ vibrissae within the TG: (**A**) Schematic diagram showing TG neurons innervating the C2, D3, and δ vibrissae; the lower-left inset is a magnified view of the yellow-boxed area; scale bar in inset: 2000 µm. (**B**) The 3D reconstruction of the TG (coronal view). (**C**) The 3D reconstruction of the TG (horizontal view). The gray dashed lines in (**C**) correspond to the 3D spatial distributions shown in (**D**–**W**), respectively. Centered on each gray dashed line, the images show 3D spatial distribution at the coronal plane level with different thicknesses. (**D**–**W**) Local 3D spatial distribution results of the TG in the regions demarcated by gray dashed lines in (**C**), showing the distribution of reconstructed neurons within the neuronal soma distribution region. Red, green, and dark blue dots represent the reconstructed somata of neurons innervating the C2, D3, and δ vibrissae, respectively; yellow represents the outline of the autofluorescence-based neuronal background; gray represents the tissue outline of the TG. A: anterior; P: posterior; D: dorsal; V: ventral; L: lateral; M: medial; V1: ophthalmic nerve; V2: maxillary nerve; V3: mandibular nerve. Scale bars: (**A**) 2000 μm and (**B**–**W**) 200 μm.

**Figure 7:**
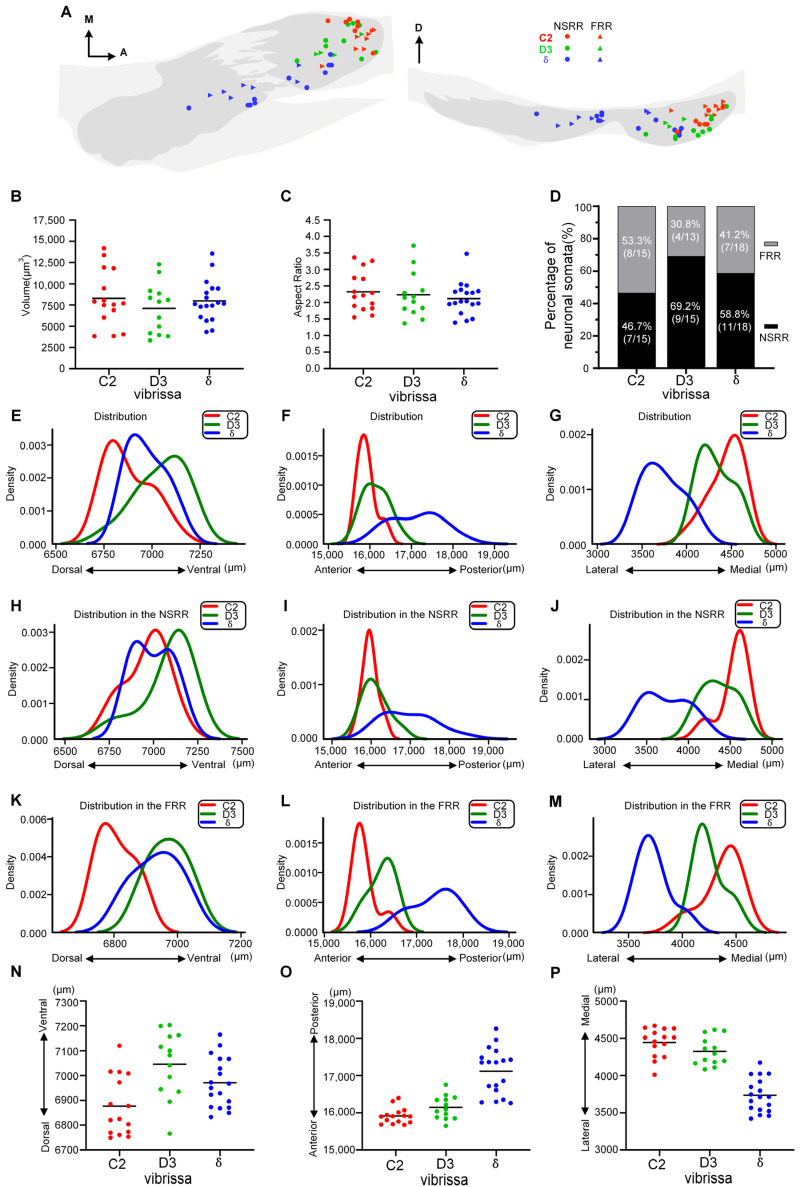
Spatial localization of reconstructed TG neurons innervating the C2, D3, and δ vibrissae along the dorsoventral, anteroposterior, and mediolateral planes: (**A**) Schematic diagram of the distribution of TG neurons innervating the C2, D3, and δ vibrissae in the TG. (**B**,**C**) Scatter plots of soma volume and aspect ratio. Each dot represents one neuron. Horizontal lines indicate mean values. These data are shown for visualization; no inferential statistical tests were performed because all reconstructed neurons were obtained from a single mouse. (**D**) Stacked bar plot showing the assignment of the reconstructed TG neurons to the operationally described NSRR and FRR. This assignment was based on the morphological description of NSRR and FRR rather than strict mask-based segmentation. (**E**–**G**) Distribution density curves of the somata of TG neurons innervating vibrissae along the three planes within the TG. (**H**–**J**) Distribution density curves of the somata of TG neurons innervating vibrissae analyzed with reference to the operationally described NSRR along the three planes. (**K**–**M**) Distribution density curves of the somata of TG neurons innervating vibrissae analyzed with reference to the operationally described FRR along the three planes. (**N**–**P**) Scatter plots showing the dorsoventral (DV), anteroposterior (AP), and mediolateral (ML) coordinates of individual neuronal somata. Each dot represents one neuron. Horizontal lines indicate mean values. These plots were used for visualization only; no inferential statistical tests were performed.

To profile the 3D spatial distributions of the TG neurons innervating the C2, D3, and δ vibrissae, we reconstructed a total of 46 TG neurons from the retrograde-labeling dataset, including 15 TG neurons innervating the C2 vibrissa, 13 TG neurons innervating the D3 vibrissa, and 18 TG neurons innervating the δ vibrissa (Figure 6A–C). In this dataset, reconstructed neurons associated with all three vibrissae were observed in both the NSRR and FRR within the TG (Figure 6D–W). To conduct an analysis of the 3D spatial distribution patterns of the TG neurons innervating vibrissae (Figure 7A), we first analyzed the morphological features, including soma volume and aspect ratio. These morphological parameters appeared broadly similar among the three groups of reconstructed neurons (Figure 7B,C). We then assigned the reconstructed neuronal somata relative to the operationally described NSRR and FRR. In this dataset, neurons from all three vibrissa groups were observed in both NSRR and FRR, and their proportions appeared comparable among the three groups (Figure 7D). This analysis was based on the morphological definition of NSRR and FRR rather than strict mask-based segmentation and was used to provide anatomical context for the reconstructed neurons.

To analyze the 3D localization of the somata of TG neurons innervating the three vibrissae, we projected the 3D distributions of these labeled TG neuronal somata onto the dorsoventral, anteroposterior, and mediolateral planes and plotted density distribution curves along the three planes. We observed that the three reconstructed neuronal populations showed considerable overlap along all three planes (Figure 7E–G). Along the dorsoventral plane, the distribution of the somata did not show clear somatotopy and showed substantial overlap (Figure 7E). Along the anteroposterior plane, the somata of TG neurons innervating the δ vibrissa were distributed over a wider area (Figure 7F). Along the mediolateral plane, the somata showed a rough mediolateral distribution trend (Figure 7G). When analyzed with reference to the operationally described NSRR and FRR (Figure 7H–M), similar overlapping distribution trends were observed in both the NSRR and FRR, without obvious NSRR- or FRR-specific segregation in this dataset. In addition, we visualized and characterized the spatial distribution of the reconstructed TG neuronal somata along the three planes based on their coordinate locations (Figure 7N–P). Overall, in this dataset, the somata of TG neurons innervating the C2 vibrissa appeared to show a dorsal shift, whereas the somata of TG neurons innervating the δ vibrissa occupied a broader anteroposterior range.

## 4. Discussion

### 4.1. Capability of fMOST-Based 3D Cytoarchitectural Mapping in Characterizing TG Anatomical Organization Compared with Traditional Methods

Previous studies using 2D histology [26,27], retrograde tracing [9,10,11], and electrophysiology [12,13,14] have provided important insights into TG architecture and vibrissa-innervating TG neurons. However, most of these methods fail to resolve continuous 3D cytoarchitectural details or only characterize labeled neurons at the population level. Recent advances in large-volume tissue processing and imaging have enabled 3D and even inter-organ imaging of large specimens, facilitating more comprehensive delineation of the anatomical correspondence between trigeminal ganglion (TG) neurons and their peripheral sensory receptors. Representative approaches combining retrograde tracers with tissue clearing have further unraveled the overall spatial topography linking these two compartments [18]. Nevertheless, current tracing strategies predominantly rely on bulk neuronal labeling, which carries the risk of nonspecific uptake at axonal terminals. More importantly, constrained by the limited resolving power of existing clearing protocols for fine anatomical subdivisions within the TG, spatial mapping analyses routinely treat the TG and its embedded sensory neurons as a homogeneous unit, failing to account for the intrinsically heterogeneous cytoarchitectural organization of the TG.

In this study, fMOST-based 3D cytoarchitectural mapping with PI staining was used in the mouse head to reveal the detailed cytoarchitectural characteristics of TG, wherein individual sensory neurons were reliably distinguished via their unique cellular morphologies. Accordingly, the authentic neuronal distribution and intrinsic cytoarchitectural patterning across the TG can be directly visualized and digitally reconstructed, permitting precise demarcation of two discrete TG subregions: the NSRR and FRR.

Furthermore, we combined retrograde viral tracing with Ai14 reporter transgenic mice to fluorescently label vibrissa-specific TG neurons. Coupled with single axon tracing, this combinatorial approach defines the precise innervation pattern between individual TG neurons and their corresponding target vibrissae. This design eliminates the labeling artifacts originating from leaky infection inherent to bulk labeling, enabling faithful characterization of the topographic mapping of whisker-selective sensory neurons within distinct TG subdomains.

Overall, this workflow provides a practical approach for cellular-resolution cytoarchitectural analysis of the TG and precise localization of target-specific sensory neurons.

### 4.2. 3D Spatial Distribution of the Distribution Regions of Somata in the TG

Our results showed that neuronal somata in the ventral region were denser and larger, while those in the dorsal region were smaller and more scattered. Krastev et al. [28] reported the opposite pattern in the human TG, where dorsomedial neurons are more densely packed and smaller than ventrolateral neurons. This difference may be related to species differences between mice and humans. The soma sizes of ganglion cells are related to their function [21,29,30]: small-diameter neurons detect noxious stimuli, and medium- to large-diameter neurons preferentially detect low-threshold mechanical stimulation. Notably, our results showed distinct morphological diversity of TG neuronal somata with region-specific distribution: round and oval somata were predominantly localized to the ventral region, and polygonal somata were also mainly distributed in the ventral region, whereas elongated somata were predominantly localized to the dorsal region. These observations may reflect cellular heterogeneity within the TG. However, because molecular or physiological markers were not included in this study, we cannot directly assign these morphologically defined neuronal populations to specific sensory modalities.

The structural heterogeneity of the TG may influence peripheral sensory signal processing through distinct subregional structural and cellular characteristics, with SGCs and SCs playing key regulatory roles. In the TG, densely packed neuronal somata are tightly enwrapped by SGCs to form distinct functional units [31]. In the TG, neuron–glia signal transmission is mediated by gap junctions: neurons activate SGCs via gap junctions, and activated SGCs in turn further activate adjacent neurons via the same pathway, thus facilitating interneuronal signal transmission [8,32]. The dense aggregation of neurons may shorten the signal transmission distance and facilitate the efficient exchange of signaling molecules. In this study, we operationally described regions enriched in neuronal somata as the NSRR. This region was used as a cytoarchitectural reference rather than as a functionally validated subregion. In contrast, the FRR was characterized by sparsely distributed neurons, abundant nerve fibers and SCs, as well as spatially isolated neuronal aggregates. Such structural features suggest that the FRR may mainly represent a fiber-dominant compartment within the TG; however, its functional role in sensory signal transmission remains to be determined.

### 4.3. 3D Distribution of TG Neurons Innervating the C2, D3, and δ Vibrissae Within the TG

Previous studies have reported that TG neurons innervating different vibrissae on the mystacial pad exhibit overlapping distributions within the TG [18,33,34]. Consistent with these previous population-level observations of vibrissa-row-dependent topography [20], our single-neuron reconstruction dataset showed that the somata of TG neurons innervating the C2, D3, and δ vibrissae showed partially overlapping yet spatially biased distributions along the dorsoventral, anteroposterior, and mediolateral planes. This observation suggests that, in the analyzed specimen, these somata of TG neurons were intermingled to some extent within the TG in 3D and did not form a strictly segregated region. This pattern differs from the more discrete topographic organization observed in central vibrissal pathways [2,3]. Thus, the present analysis extends previous observations using single-neuron reconstruction, rather than establishing a statistically generalized somatotopic map of the TG.

The δ vibrissa is a special type of vibrissa, known as a straddler vibrissa. Although straddler and regular vibrissae show no fundamental difference in tactile coding [13,35], previous studies have reported different TG distribution patterns for neurons innervating different vibrissae. For example, the TG neurons innervating the γ vibrissa display a widespread distribution in the ophthalmic–maxillary part of the TG [36], while the TG neurons innervating the B1 and B2 vibrissae show a clustered pattern [34]. In this study, neurons innervating the δ vibrissa occupied a comparatively broader spatial extent along the anteroposterior plane than TG neurons innervating the C2 and D3 vibrissae. Neurons with broader soma distributions in the TG have been reported to possess larger axonal projection fields in the brainstem and may be more likely to form complex central signal transmission circuits [37].

When analyzed with reference to the operationally described NSRR and FRR, TG neurons innervating the C2, D3, and δ vibrissae were observed in both regions, and similar overlapping trends were present in both NSRR and FRR. This exploratory subregional analysis suggests that the operationally described soma-rich and fiber-rich regions provide additional anatomical context for describing labeled neuronal distributions, but no obvious NSRR- or FRR-specific segregation was observed in this dataset.

### 4.4. Limitations and Prospects

Although this study demonstrates the practical applicability of the fMOST-based workflow for 3D cytoarchitectural analysis of the mouse TG and single-neuron localization of TG neurons innervating vibrissae, several methodological limitations should be acknowledged. First, all reconstructed TG neurons innervating the C2, D3, and δ vibrissae were derived from a single mouse. Therefore, the present dataset lacks biological replication, and no inferential statistical analysis based on biological replicates was performed. Accordingly, the spatial distribution of the reconstructed neurons is described only as an anatomical observation within the analyzed specimen. Future studies including multiple animals will be required to determine whether similar distributions can be observed in independent biological samples.

Second, the throughput of the current workflow remains limited. Whole-head high-resolution fMOST imaging preserves the spatial continuity among the mystacial pad, TG, and brain but generates large-volume datasets that require substantial storage, processing, visualization, and manual reconstruction. In addition, retrograde viral labeling and single-neuron tracing require sparse and sufficiently bright signals; relatively weak tdTomato expression in Ai14 mice and bright signals near the injection sites may limit the complete tracing of distal axonal terminals within the follicle–sinus complex. These technical constraints currently restrict the number of samples, vibrissae, and reconstructed neurons that can be analyzed. Future improvements in labeling strategies, image processing, and semi-automated or automated reconstruction tools will help increase the efficiency and throughput of this workflow.

## 5. Conclusions

Based on the findings presented in this study, the following conclusions can be drawn:

By applying the fMOST imaging combined with PI staining and reconstruction workflow, we acquired the cellular-resolution 3D cytoarchitectural information of the mouse TG. Based on heterogeneous spatial patterning of intrinsic sensory neurons, we precisely delineated two morphologically divergent TG subdomains, termed NSRR and FRR. Using retrograde genetic viral labeling coupled with single-axon tracing to verify vibrissa-specific innervation, we further mapped the subregional three-dimensional positioning of TG neurons innervating the C2, D3, and δ vibrissae. These reconstructed neuronal populations were observed in both NSRR and FRR and showed partially overlapping yet spatially biased distributions along the dorsoventral, anteroposterior, and mediolateral axes of the TG, consistent with prior population-scale findings of row-dependent vibrissal topography. Compared with C2 and D3 vibrissae, TG neurons targeting δ vibrissa occupied a wider anteroposterior spatial range.

Collectively, this study demonstrates the practical applicability of the present fMOST-based imaging and reconstruction workflow for 3D cytoarchitectural analysis of the mouse TG and single-neuron localization of target-related sensory neurons. These high-resolution 3D morphological data and anatomical observations establish a reliable reference framework for future investigations of the trigeminal system.

## Data Availability

The original contributions presented in this study are included in the article. Further inquiries can be directed to the corresponding author.

## Abbreviations

The following abbreviations are used in this manuscript:

2D	two-dimensional
3D	three-dimensional
BMA	butyl methacrylate
CNS	central nervous system
DAPI	4′,6-diamidino-2-phenylindole
DVN	deep vibrissal nerve
fMOST	fluorescence micro-optical sectioning tomography
FRR	fiber-rich region
FSC	follicle–sinus complex
GMA	glycidyl methacrylate
NSRR	neuronal soma-rich region
ORS	outer root sheath
PBS	phosphate-buffered saline
PFA	paraformaldehyde
PI	propidium iodide
Pr5	principal sensory nucleus
S1	primary somatosensory cortex
SCs	Schwann cells
SGCs	satellite glial cells
Sp5O	spinal trigeminal nucleus, oral part
TG	trigeminal ganglion
V1	ophthalmic nerve
V2	maxillary nerve
V3	mandibular nerve
